# Distance tuneable integral membrane protein containing floating bilayers *via in situ* directed self-assembly†

**DOI:** 10.1039/d3nr04622b

**Published:** 2024-06-24

**Authors:** Stephen C. L. Hall, David J. Hardy, Éilís C. Bragginton, Hannah Johnston, Tudor Onose, Rachel Holyfield, Pooja Sridhar, Timothy J. Knowles, Luke A. Clifton

**Affiliations:** a ISIS Pulsed Neutron and Muon Source, Science and Technology Facilities Council, Rutherford Appleton Laboratory Harwell Science and Innovation Campus Didcot Oxfordshire OX11 OQX UK luke.clifton@stfc.ac.uk; b School of Biosciences, University of Birmingham Birmingham B15 2TT UK; c Electron Bio-Imaging Centre (eBIC), Diamond Light Source Ltd Diamond House Harwell Science and Innovation Campus OX11 0DE UK

## Abstract

Model membranes allow for structural and biophysical studies on membrane biochemistry at the molecular level, albeit on systems of reduced complexity which can limit biological accuracy. Floating supported bilayers offer a means of producing planar lipid membrane models not adhered to a surface, which allows for improved accuracy compared to other model membranes. Here we communicate the incorporation of an integral membrane protein complex, the multidomain β-barrel assembly machinery (Bam), into our recently developed *in situ* self-assembled floating supported bilayers. Using neutron reflectometry and quartz crystal microbalance measurements we show this sample system can be fabricated using a two-step self-assembly process. We then demonstrate the complexity of the model membrane and tuneability of the membrane-to-surface distance using changes in the salt concentration of the bulk solution. Results demonstrate an easily fabricated, biologically accurate and tuneable membrane assay system which can be utilized for studies on integral membrane proteins within their native lipid matrix.

## Abstract 2

Surface distance tuneable integral membrane protein containing floating bilayers produced by *in situ* directed self-assembly. Bio-mimetics which allow for membrane protein studies within the lipid matrix under biologically accurate conditions.

## Graphical abstract

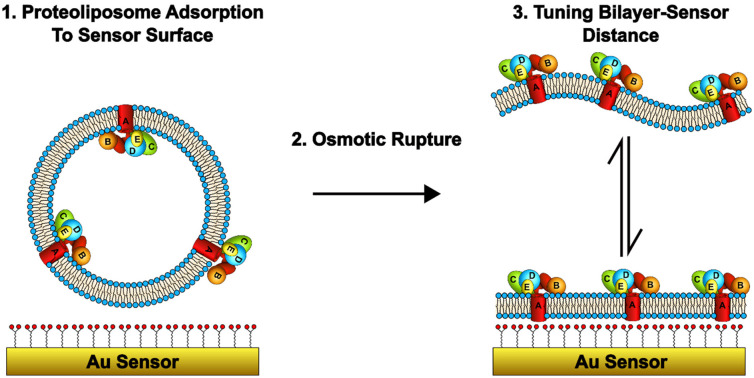

## Introduction

Membranes are the key structural material of biology at the cellular level. These complex assemblies of mostly lipid and protein define what is and what is not of the cell as well as the many organelles which carry out cellular function. Examining membrane biochemistry at the molecular level is challenging due to the immense compositional complexity of membranes as well as their thin size in the transverse direction (∼6 nm). To address this, model membranes have become vital *in vitro* tools for gaining a precision molecular level understanding of membrane relevant biochemical events.^1^

The protein component of the membrane is subdivided into integral (membrane embedded) and peripheral (membrane bound) proteins. Integral membrane proteins (IMPs) make up around one third of the mass of a biological membrane^2^ and ∼23% of the human proteome.^3^ IMPs are key to cellular function and are involved in signal transduction, energy conversion, cell transport and cell–cell interactions to name a few of their many roles. The importance of IMPs in cellular processes means that ∼60% of drug targets are proteins from this class.^4–6^*In vitro* studies on membrane proteins are extremely challenging due to their amphiphilic nature and IMP stability problems outside of their native membrane environment. To combat this many *in vitro* approaches have been developed to examine IMPs such as the extraction of these into membrane mimetics such as detergents,^7^ amphipols^8^ and lipid nanodiscs.^9,10^ Despite the development of such approaches, studying IMPs *in vitro* in their native membrane environment remains problematic. Giess *et al*.^11^ developed a method for reconstituting IMPs into a planar membrane by tethering histidine-tagged proteins onto a self-assembled monolayer (SAM) surface, allowing subsequent reconstitution of a lipid bilayer around this *via* exchange of solubilising detergent for lipids, creating a single planar membrane. This methodology is very useful though requires engineering of a non-native sequence on either membrane proximal or distal side of the IMP.

We and others have been developing planar floating lipid bilayers which sit adjacent to bulk interfaces but are not in contact with them.^12–14^ There are numerous advantages of such samples over other membrane models such as supported or tethered planar lipid bilayers which are in close proximity or anchored to a bulk surface. Floating supported bilayers have improved biological accuracy as they allow for greater fluctuations of the membrane,^15^ they have significant water reservoirs on both sides of the bilayer^16^ and have reduced surface influence on the lipid behaviour.^17^ When combined with the myriad of interfacial analytical techniques which can probe such samples, floating bilayers are biologically accurate membrane mimetics which are amenable to precision molecular level characterization.

Recently, we simplified the fabrication of such sample systems by developing an *in situ* self-assembled floating lipid bilayer sample system^17,18^ formed by incubating lipid vesicles in the presence of a carboxyl terminated oligoethyleneglycol alkane thiol self-assembled monolayer (COOH-OEG-SAM) coated gold surface followed by a temperature ramp. This methodology means floating supported bilayers can be fabricated without the need of specialist Langmuir–Blodgett troughs and time consuming, technically challenging layer-by-layer deposition methodologies.^13,14^

We discovered that altering buffer salt conditions around the self-assembled floating supported bilayers could be used to reversibly tailor the distance between the COOH-OEG-SAM and the membrane.^17,18^ The bilayers were found to be closest to the COOH-OEG-SAM surface (∼10–15 Å away) in the presence of Ca^2+^ ions. The removal of these by EDTA caused the membrane to reversibly move >200 Å from the SAM surface. The replacement of the divalent calcium with monovalent ions at physiological concentrations or monovalent/divalent mixtures caused more subtle movements of the membrane from the bulk interface. Molecular dynamics simulations revealed the reasons for this behaviour were due to the accumulation of ions in the water interlayer between the COOH-OEG-SAM and the lipid bilayer both screening electrostatic repulsion between the anionic groups on the bilayer and the COOH-OEG-SAM and potentially causing cation bridging between these.^17^

Here, we demonstrate how self-assembled floating supported protein-lipid membranes containing a large multi-domain IMP complex, namely the Gram-negative bacterial β-barrel assembly machinery (Bam) complex,^19,20^ can be fabricated using a simple two-step vesicle surface binding and osmotic shock methodology. Furthermore, we show that the IMP containing membranes distance from the surface can be reversibly tuned to the experimental requirements through changes in solution salt conditions around physiological values. This sample system therefore has potential as an easily fabricated, tuneable and biologically accurate membrane mimetic for studies on IMPs in their native lipid matrix.

## Materials and methods

### Materials

POPC (1-Palmitoyl-2-oleoyl-*sn-glycero*-3-phosphocholine) and POPS (1-Palmitoyl-2-oleoyl-*sn-glycero*-3-phospho-l-serine) were obtained from Avanti polar lipids (Alabaster, AL, USA) and used without further purification. HS-C_11_-EG_3_-OCH_2_-COOH (COOH-OEG-SAM) was obtained from Prochimia surfaces (Gdansk, Poland). Deuterium oxide (D_2_O), TRIS base and HEPES buffer salts and all other chemicals were sourced from Sigma-Aldrich (St Louis, USA) or Fisher Scientific (Loughborough, UK). Silicon substrates were obtained from Crystran (Poole, UK).

### Membrane protein purification and preparation of protein–lipid vesicles

BamABCDE was purified as detailed by Roman-Hernandez *et al*.^21^ Briefly, *E. coli* strain BL21(DE3) (New England Biolabs) transformed with plasmid pJH114 (kindly provided by H. Bernstein) was grown overnight at 37 °C in LB containing 100 μg mL^−1^ ampicillin (Melford laboratories). 10 mL of the overnight culture was added to 1 L of LB containing 100 μg ml^−1^ ampicillin. When the cultures reached OD_600_ = 0.5–0.6, 0.4 mM IPTG was added to induce the expression of BamABCDE and the culture was incubated for 1.5 hours at 37 °C. The cells were then centrifuged at 5000*g* for 15 minutes at 4 °C (JLA 8.1000 rotor, Beckman Coulter, JXN26 centrifuge). Cell pellets were resuspended in 10 mL L^−1^ of cold 20 mM Tris–HCl pH 8, 150 mM NaCl and the cells were lysed using an EmulsiFlex C3 cell disruptor (Avestin). Lysates were centrifuged at 10 000*g* (JA25.50, Beckman Coulter, JXN26 centrifuge) at 4 °C for 30 minutes. The supernatants were centrifuged in a Ti 70 rotor (Beckman Coulter) for 60 minutes at 100 000*g* at 4 °C for 1 hour, to harvest the membranes. The membrane pellets were homogenised (1 mL per 40 mg of membrane) and incubated in cold 50 mM Tris pH 8, 150 mM NaCl, 1% (w/v) N-dodecyl β-d-maltoside (DDM) on ice for 1 h and the centrifugation step was repeated. Supernatants containing the solubilised membrane proteins were then rotated in the presence of 2 mL L^−1^ Ni-NTA agarose (Qiagen) for 1.5 h at 4 °C. Ni-NTA beads were washed with one column volume of 50 mM Tris pH 8, 150 mM NaCl, 0.03% DDM, 50 mM imidazole. BamABCDE was then eluted in 5 mL of the above buffer containing 500 mM imidazole and injected onto a S200 column (Cytiva) equilibrated with 50 mM Tris pH 8.0, 150 mM NaCl, 0.03% DDM. The column was run at 0.5 mL min^−1^ and 1 mL fractions were collected. Fractions that contained complete BamABCDE complexes were identified by SDS-PAGE (Genscript, Bis–Tris gels), pooled and concentrated to 1 mg mL^−1^.

Proteoliposomes were formed by incubating 1 mg of purified BamABCDE with 10 mg POPC : POPS (8 : 2 mol : mol) for half an hour on ice. The sample was then spun at 13 500*g* for 10 minutes to pellet any insoluble material and the supernatant purified through an S75 10 300 column (Cytiva). The fractions containing the proteoliposomes were pooled and the concentration determined *via* UV.

### Permalloy/gold coating of silicon crystals

Piranha acid cleaned silicon crystals (50 × 80 × 15 mm) with a polished 80 × 50 mm face (111 orientation, surface roughness (RMS) ∼3 Å) were sequentially sputter coated with Permalloy (Ni_80_Fe_20_) and gold at the NIST centre for Nanoscience and Technology, Gaithersburg, MD, USA, in a Denton Discovery 550 sputtering chamber (Denton Vacuum, New Jersey, USA)_._

### Self-assembled monolayer coating of gold surfaces

Permalloy and gold coated silicon substrates were cleaned with a 1% solution of Hellmanex™, followed by washing with ultrapure H_2_O (upH_2_O) and absolute ethanol (99.8% purity). These were then dried under nitrogen before UV-Ozone cleaned for 20 minutes (T10X10 model from UVOCS, Pennsylvania, USA), washing with upH_2_O, drying again under nitrogen and again cleaning with ozone.

The cleaned surface was then fully submerged in absolute ethanol solution containing ∼70 μM COOH-OEG-SAM in sealed containers and incubated for 48 hours at room temperature under low light conditions. After this time the substrates were removed from the solution, washed with ethanol, upH_2_O and then sonicated in a 1% solution of sodium dodecylsulfate (SDS) before washing again with ethanol and upH_2_O and dried under nitrogen.

The presence of the COOH-OEG-SAM on the gold surface was checked using a rudimentary (*i.e.* by eye) contact angle assessment of 1 μL of upH_2_O of the SAM coated gold surface (being ∼25° for a coated surface).

**Neutron Reflectometry** (NR) was employed to analyse the structure of the self-assembled floating supported protein-lipid membranes adjacent to the COOH-OEG-SAM on Permalloy and gold coated silicon surbstrates. NR measurements were carried out using the white beam SURF^22^ and INTER^23^ reflectometers at the ISIS Spallation Neutron Source, Rutherford Appleton Laboratory (Oxfordshire, UK), which use neutron wavelengths from 0.5 to 7 Å and 1 to 16 Å respectively. The reflected intensity was measured at glancing angles of 0.35°, 0.65° and 1.5° for SURF and 0.7° and 2.3° for INTER. Reflectivity was measured as a function of the momentum transfer, *Q*_*z*_ (*Q*_*z*_ = (4π sin *θ*)/*λ* where *λ* is wavelength and *θ* is the incident angle). Data were obtained at a nominal resolution (*dQ*/*Q*) of 3.5%. The total illuminated sample length was ∼60 mm on all instruments.

Details of the solid-liquid flow cells and liquid chromatography setup used in the experiments described here are described in a previous article by us.^24^ Briefly, the COOH-OEG-SAM coated gold surfaces were assembled into submerged bespoke solid–liquid flow cell and sealed. The solid liquid flow cells were then placed onto the instrument sample position and connected to instrument controlled HPLC pumps (Kauer Smartline 1000) which controlled the change of solution isotopic contrast in the flow cell as well as change in solution counter ion concentration and type. The samples were aligned parallel to the incoming neutron beam with the beam width and height controlled using two collimating slits prior to the sample position. The sample height was aligned in such a way that the neutron beam was centred on the middle of the sample surface.

#### Surface structure measurements

Initially measurements of the COOH-OEG-SAM/gold/permalloy coated substrates were conducted by measuring NR data under two solution isotopic contrast conditions (H_2_O and D_2_O buffer conditions). Upon confirmation of COOH-OEG-SAM coating on the gold surfaces with suitable surface coverage (≥90%), floating bilayer fabrication then took place.

#### Protein–lipid membrane deposition

**Fig. 1:**
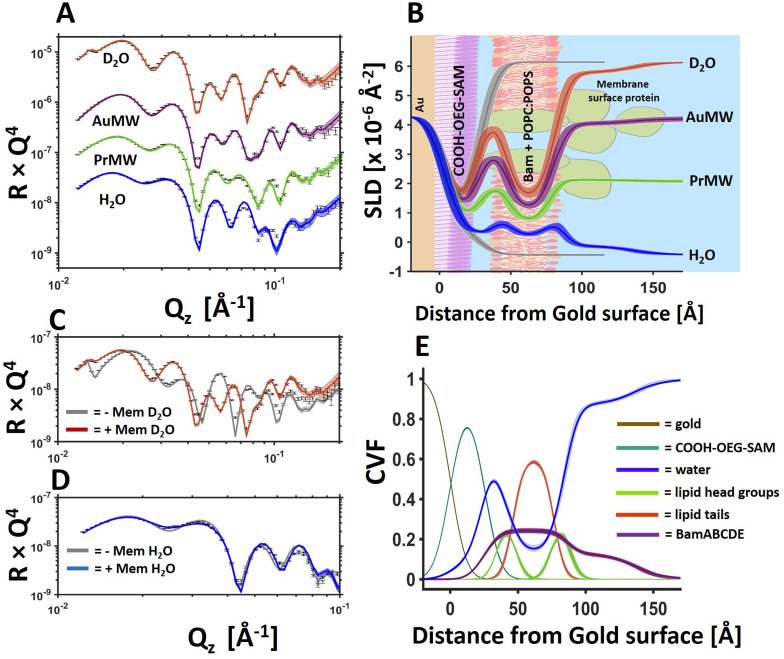
BamABCDE in a floating 8 : 2 (mol mol^−1^) POPC : POPS bilayer adjacent to a COOH-OEG-SAM at the gold/water interface. Neutron reflectometry (NR) profiles (error bars) and model data fits (lines) under multiple solution isotopic contrast conditions for the protein–lipid membrane containing sample (A) and the scattering length density profiles of the gold/water interfacial region are shown with a schematic of the proposed interfacial structure superimposed (B). The sample was measured in 20 mM HEPES buffer pH/D 7.2 with 100 mM NaCl 2 mM CaCl_2_. A comparison of the NR profiles before and after the deposition of the floating membrane in D_2_O (C) and H_2_O (D) are shown to highlight the modulation of the NR profile by the membrane. Finally, the component volume fraction profile of the gold water interfacial region determined by NR data analysis is given, showing the relative distribution of the SAM, water, lipid and protein components (E). The range of acceptable fits used to generate the 65% confidence intervals for the fitting parameters are shown as a line width in A, C and D and the ambiguity in the resolved interfacial structure determined from this are shown as line widths in B and E. The fabrication of this sample was repeated in duplicate with the data and fits for one of the replicate depositions shown in Fig. 3 and in ESI Fig. 3.†

Floating planar IMP-lipid membranes were found to be fabricated *via* vesicle rupture by osmotic shock. In this methodology a 0.1 mg mL^−1^ suspension of vesicles of 1 : 10 (w/w) BamABCDE: 8 : 2 (mol mol^−1^) POPC : POPS in 20 mM Tris–HCl, 2 mM CaCl_2_, 200 mM NaCl, pH/D 7.2 were flushed into a solid liquid flow cell containing a COOH-OEG-SAM coated gold surface. The sample was incubated for ∼30 minutes after which time a non-buffered solution of 2 mM CaCl_2_ was flushed through the solid/liquid flow cells followed by 20 mM Tris–HCl, 2 mM CaCl_2_, 200 mM NaCl, pH 7.2. The formation of the resulting protein–lipid planar floating supported membrane was then either analysed structurally with NR (Fig. 1) or through the changes in coupled mass at the gold/water interface by QCM-D (Fig. 5).

#### NR data analysis

NR data were analysed using RasCal 2019^25^ running under Matlab (version 2020b). This software uses optical matrix formalism^26^ to fit layer models describing the neutron scattering length density (SLD) profiles across bulk interfaces to the experimental NR datasets and is particularly focused towards simultaneously fitting multiple differentially isotopically labelled NR data sets (termed contrasts).

The data from the protein–lipid floating membranes were fitted using the custom model option in RasCal. In this approach the relationship between the fitted structural parameters (SAM and membrane coverage, lipid and protein distribution relative to the gold surface) and the scattering length density profile used to generate the model NR data sets are described, as are the relationships between the individual isotopic contrast data sets. Genetic and least squares fitting algorithms were used to optimize the agreement between the experimental and model data sets.

In general, four individual NR data sets were simultaneously fitted for each structure/experimental condition. These data sets were the samples measured under differing buffer solution isotopic contrast conditions, being 100% D_2_O, gold matched water (AuMW, 75% v/v D_2_O), protein matched water (PrMW, 42% D_2_O v/v) and 100% H_2_O. In addition to this, two additional data sets which were collected before the membrane deposition, were simultaneously fitted with the same “under-layers” (see definition below) to additionally constrain the interfacial structure and help gain a unique solution to this.

In the custom model describing the interfacial structure the under-layers (the layers between the silicon surface and the gold/water interface) were, moving from the silicon substrate to the solution subphase, a mixed silicon dioxide/permalloy layer adjacent to the silicon substrate, a permalloy layer next to this, a gold layer and the COOH-OEG-SAM layer. This under-layer structure was simultaneously fitted across all data sets (with and without the protein lipid membrane) and across all isotopic contrasts.

In data sets collected with the protein–lipid membranes present at the SAM/water interface an additional multilayer structure was added to the model to represent the membrane. This included a layer of water between the SAM and the floating membrane and the layers describing the protein–lipid membrane itself. These consisted of an inner protein distribution composed of protein and water only, an inner head group region composed of lipid head groups, protein or water, a lipid tail region composed of lipid tails, protein or water, an outer head group region similar to the inner head group region and an extra-membranous distribution of protein on the surface of the membrane facing the bulk solution (layer composed of protein and water). The ratio of protein/lipid and water in the lipid bilayer region of the membrane was fitted in such a way that the total volume fraction of the components could not exceed nor be less than 100%.

Bayesian inference of the ambiguity of the resolved structures from NR model-to-data fitting was undertaken using MCMCStat (https://mjlaine.github.io/mcmcstat) Delayed-Rejection Adaptive Metropolis algorithms (DRAM)^27^ Monte–Carlo–Markov Chain (MCMC) routines to refit the data using a user defined number of steps. To fit data using this approach, the likelihood function is defined in terms of the Chi-squared goodness of fit criteria, as shown previously.^28^ The parameter uncertainties were then determined from the posterior distributions as the shortest percentile confidence interval from each (in this case 65%) and the uncertainties on the reflectivity's and SLD's were generated by randomly sampling (in this case 1000 samples) from the Markov chains, calculating reflectivity's and SLD's for each set of samples, and taking the relevant percentile across all the resampled reflectivity or SLD curves at each point in *Q*_*z*_ (or distance) to represent the uncertainties on the fits. The chain samples are also used to generate the line shading used to denote the ambiguity of the structure across the interface in the volume fraction profiles. Best fit lines are the mean of the reflectivity and SLD uncertainties and are shown as a darker line in figures.

Finally, component volume fraction *vs.* distance profiles were generated using custom scripts which took the model description in the rascal custom model and the parameter distributions to construct the relative distribution of each component across the interface in term of volume fraction. Rascal's boxcar function routine used to build the layer structure was used to calculate the relative volume fraction and error of each component over each 1 Å interval across the solid/liquid interface (*z* axis) using the component volume fraction, layer thicknesses and roughness's as inputs. The water distribution across the solid–liquid interface was calculated as the unoccupied volume.

#### NR Studies on BamABCDE activity in floating supported bilayers

To assess Bam activity, protein–lipid self-assembled floating membranes were deposited 1 : 10 (w/w) BamABCDE: 8 : 2 (mol mol^−1^) POPC : POPS in 20 mM HEPES buffer pH/D 7.2 with 100 mM NaCl 2 mM CaCl_2_ onto the COOH-OEG-SAM coated gold surfaces using the same osmotic shock procedure as before. Activity was then assessed by measuring the impact of incubation with a client/chaperone complex. This was performed using the unfolded client outer membrane protein T (uOmpT) and chaperone SurA. Per-deuterated (d-)uOmpT and SurA were expressed and purified as detailed in Hall *et al.*^20^ D-uOmpT, as an inclusion body, was resuspended in 8 M urea and diluted into a solution of 4 μM SurA in 20 mM Tris, 100 mM NaCl, 2 mM CaCl_2_, pH 8 to give a final d-uOmpT concentration of 0.4 μM in 800 mM urea. The sample was then incubated for 30 minutes on ice then centrifuged at 5000*g* for 5 minutes prior to addition to the flow cell. The solutions were hand injected into the NR flow cells and incubated for 270 minutes at 37 °C before final NR analysis of the membrane structure after the removal of excess SurA/d-uOmpT.

#### QCM-D analysis

Quartz crystal microbalance measurements were undertaken on a Q-Sense QE4 instrument (Biolin Scientific, Västra Frölunda, Sweden) which allows for the simultaneous analysis of the changes in oscillating frequency and dissipation of up to four individual sensor surfaces.

Q-sense gold QCM-D sensors were cleaned with a 1% Hellmanex solution, followed by washes in upH_2_O and EtOH before drying under a stream of nitrogen. The surfaces were then cleaned with UV-Ozone (T10X10 model from UVOCS, Pennsylvania, US), washed again with upH_2_O to remove any surface ash before cleaning with UV-Ozone again. The sensors were then incubated in a 70 μM solution of COOH-OEG-SAM in HPLC grade ethanol for 48 hours at room temperature under low light conditions.

After incubation the sensors were washed with ethanol and sonicated in a solution of 1% SDS before washing with upH_2_O and ethanol and dried under a stream of nitrogen before mounting into the QCM instrument's solid/liquid flow cells. Here, three cells were simultaneously analysed for each experiment. Two being used for the examination of protein–lipid membrane samples on COOH-OEG-SAM surface and another being a bare COOH-OEG-SAM surface measured under the same changing solution conditions used as a control.

Protein–lipid floating membranes were deposited onto the sensor surfaces using the same osmotic shock procedure used for the samples examined by NR with the only difference being the use of HEPES rather than Tris buffer salts.

Upon deposition of the samples, the solution salt conditions were changed to check the distance tuning activity of the resulting floating membrane sample.^17^ Decreases in frequency and/or increases in dissipation are associated with increasing membrane-to-SAM distance. These correlations, discovered *via* a direct comparison between QCM-D and NR results from our previous work,^17^ come from the fact that QCM measures changes in coupled mass at the surface.^29,30^ Therefore, water trapped between the SAM and the membrane will contribute to the measured frequency and dissipation changes as long as the membrane is still correlated with the surface.^30^ The depth sensitivity of QCM-D measurements is inversely proportional to the overtone measured. In pure water at 38 °C we estimate a penetration depth of 120 nm, 94 nm and 79 nm for the 3^rd^, 5^th^ and 7^th^ overtones respectively for the standard gold Q-Sense QCM sensors used.^31^ This range from the surface is well within the range of membrane-to-surface distances found for the self-assembled floating bilayers by us previously.^17^ However, it is suggested here that at very large distances (such as those found in the presence of EDTA in the buffer solution) the floating membrane may fully or partially decouple from the surface due to the presence of bulk water between the SAM and this leading to a frequency increase/dissipation decrease (see Fig. 5).

#### CryoEM sample preparation and data collection

Four microliters of freshly prepared proteoliposomes at a protein concentration of 0.5 mg ml^−1^ were deposited onto glow discharged R1.2/1.3 Cu 300 mesh holy carbon grids (Quantifoil) prior to vitrification. Grids were plunge frozen using a Virtobot Mark IV (Thermo Fisher Scientific) (Blot force 2, Blot time 3 s, temperature 22 degrees) and stored in liquid nitrogen prior to imaging. Data was collected at the electron Bio-Imaging Centre (eBIC) using a Glacios microscope (Thermo Fisher Scientific) equipped with a field emission gun operating at 200 keV and a Falcon 4 direct electron detector (Thermo Fisher Scientific). Data was collected with EPU software (Thermo Fisher Scientific) using a defocus range of −3 to −5 μm at a magnification of ×120 000 with a corresponding pixel size of 1.212 Å per pixel. The total dose applied to the sample was 45 e Å^−2^.

#### Dynamic light scattering of protein lipid vesicles

Dynamic light scattering (DLS) experiments were performed using Nanosizer S diffraction particle sizer (Malvern Instruments, UK) with a 5003 multi-digital correlator. The light source was a 2 mW He–Ne laser, linearly polarized, with *λ* = 633 nm, and scattering angle where *θ* = 173°.

## Results and discussion

Following on from our previous work on the development of *in situ* self-assembled floating lipid bilayers,^32^ we wished to investigate whether the same sample system could be developed further for the study of IMPs in contiguous planar mimics of their native lipid matrix. Using a simple osmotic shock procedure, self-assembly of IMP containing proteo-liposomes into a floating membranes was observed at the gold/water interface. In this methodology protein–lipid vesicles were re-suspended in a buffer containing 150 mM NaCl and 2 mM CaCl_2_ and incubated in the presence of a COOH-OEG-SAM coated gold surface where the vesicles adsorbed adjacent to the SAM.^18^ Following this a solution of 1 mM CaCl_2_ (only) was flushed over the surface causing the vesicles to rupture, forming a floating planar membrane. Upon two repeated attempts this method was found to consistently produce planar protein–lipid membranes adjacent COOH-OEG-SAM coated gold surfaces when analysed by NR (see Fig. 1 for one example, Fig. 3 and ESI Fig. S3† for another).

Fig. 1 shows an example of a protein–lipid floating membrane produced. This sample was fabricated from vesicles composed of 90% by weight of 8 : 2 mol mol^−1^ POPC : POPS lipid and 10% by weight of the full BAM complex. The structure and macromolecular complexity of the resulting model membrane system was resolved using NR. Revealing the lipid tails region of the floating membrane were found to contain (by volume) ∼61% lipid, 24% protein (by chain) and ∼15% water (Fig. 1E and Table 1). This water component is likely to be predominantly protein associated and found within the β-barrel of membrane spanning Bam A.^33^ On the outer surface of the membrane facing the bulk solution there was a distribution of protein which was likely to be the membrane surface periplasmic domains of Bam, namely the POTRA domains of BamA and B, C, D and E proteins. Indeed, from the asymmetry in the protein distribution across the floating membrane it seems likely that the protein component is orientated relative to the SAM surface after membrane deposition. This is supported by the observation that the protein distribution across the floating bilayer (Fig. 1E, purple line) is consistent with that of BamABCDE observed using tethered membranes.^20^ In that study BamABCDE was tethered to a Cu-NTA derived SAM surface *via* a histidine tag located within an extracellular loop of BamA so that the complex was orientated such that BamABCDE's periplasmic domains were located on the outer surface of the lipid bilayer facing the bulk solution.

**Table 1:** Resolved structural parameters from a self-assembled floating 8 : 2 POPC : POPS bilayer with embedded BamABCDE

Layer	Thickness	Composition	Roughness
COOH-SAM	25.0 (−0.5, +0.5) Å	92 (−1, +1) % SAM	9.0 (−0.5, +0.5) Å
		8 (−1, +1) % solution	
Solution interlayer	11.0 (−0.7, +0.7) Å	100% solution	7.0 (+1.0, −1.0) Å
Inner head groups	8.0 (+ 0.6, −0.6)	24 (−1, +1,) % protein	
		42 (−3, +4) % lipid	
		34 (−4, +3) % solution	
Tails	30.0 (−0.5, +0.5) Å	24 (−1, +1,) % protein	
		61 (−2, +2) % lipid	
		15 (−2, +2) % solution	
Outer head groups	8.0 (+ 0.6, −0.6) Å	24 (−1, +1,) % protein	
		42 (−3, +4) % lipid	
		34 (−4, +3) % solution	
Peripheral protein	50.5 (−3.7, +3.5) Å	13 (−1, +1) % protein	16.5 (−3.5, +3.2) Å
		87 (−1, +1) % solution	

Recently, we demonstrated that protein–lipid vesicles of the membrane surface *E. coli* Paraquat Inducible domain C and phospholipid could be co-deposited onto silicon surfaces and in the resulting structure it was found that the protein was located entirely on the distal (solution facing) side of the resulting planar membrane.^34^ This result suggests the interaction of the lipid with the surface during planar membrane formation excludes the protein forcing it onto the outside surface of the lipid bilayer. This data provides an explanation for the orientation of Bam across the floating membranes observed here. It was previously demonstrated by us that there is an attraction between phospholipids in the self-assembled floating supported membranes and the COOH-OEG-SAM^17^ coated surface. Therefore, the orientation of the Bam complex in the floating membranes maybe due to the exclusion of the periplasmic domains of the Bam complex from the water interlayer between the protein–lipid membrane and the SAM due to this attraction, forcing the protein to orientate with the largest soluble region in facing the bulk solution.

The volume fraction of protein found across the lipid bilayer region of the floating membranes in the two samples measured (being 23% and 11%, the data from which is shown in Fig. 1 and ESI Fig. S3† respectively) is greater than that found in the vesicles used to fabricate the floating bilayers. In this study a 10 : 1 w/w lipid-to-protein ratio in the vesicles was used. As the B, C, D and E components of the complex are extra-membranous with the transmembrane component being BamA we estimate the BamA to lipid ratio in the vesicles to be 23 : 1. This gives a membrane volume fraction of 97% lipid and 3% protein in the vesicles. The reason for this disparity between the vesicle and floating membrane protein-to-lipid ratio is unknown but may arrive from attractive interactions between the protein and the SAM during the vesicle adsorption stage of the deposition process enriching the protein component in the resulting floating membrane after vesicle rupture.

An important feature of the floating membrane samples is the identifiable presence of water on both sides of the planar membrane model. In the case of the lipid only samples studied previously, the thickness of the water interlayer between the SAM and the lipid bilayers was found to be between 10–15 Å in the presence of 2 mM Ca^2+^ only.^17^ Similar distances were observed with the BamABCDE : POPC : POPS floating membranes (∼11 Å, see Table 1).

**Fig. 2:**
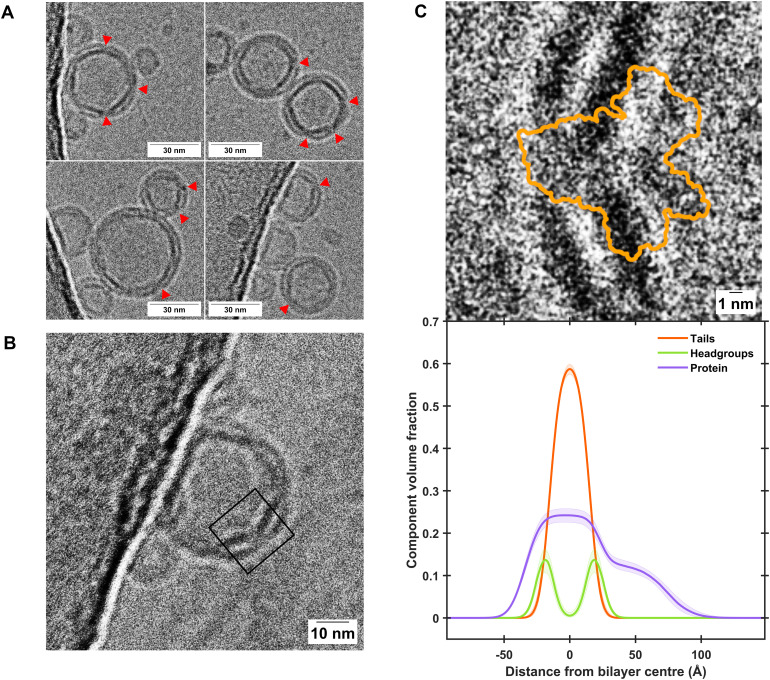
Cryo-EM characterisation of BamABCDE-containing proteoliposomes shows close agreement with component distribution determined by NR. (A) Representative excerpts of micrographs showing proteoliposome sizes of consistently less than 50 nm where clear incorporation of transmembrane proteins was observed, as indicated by red arrows. Further examples are shown in Fig. S5, and uncropped micrographs are shown in Figs. S6 and S7 of the ESI.† (B) A clear example where extramembranous domains of the Bam complex can be observed within a proteopliposome. The black box represents the region of interest which was cropped and rotated by 50° in C. (C) A comparison of the Cryo-EM image of the Bam complex within proteoliposomes (top panel) and the Component Volume Fraction distribution across a floating protein-lipid membrane determined by NR (bottom panel), showing close agreement between the two techniques. Both panels are shown on the same scale for comparison. The orange outline in the top panel depicts the extent of the BamABCDE structure as determined by X-ray diffraction (PDB ID: 5AYW).^35^

Fig. 2 gives a comparison between the volume fraction distribution of the Bam complex across the self-assembled floating bilayers with the distribution of the protein across the POPC : POPS bilayers determined from EM images of the protein–lipid vesicles used to deposit the floating membranes. This was conducted to identify if the full protein complex found within the vesicles was transferred to the protein–lipid self-assembled floating bilayers. Results revealed the length scales of the membrane spanning and peripheral membrane regions of the protein found by NR analysis of the self-assembled floating supported membranes matches that identified in the EM images (Fig. 2C), suggesting the protein structure was not altered during the deposition process.

**Fig. 3:**
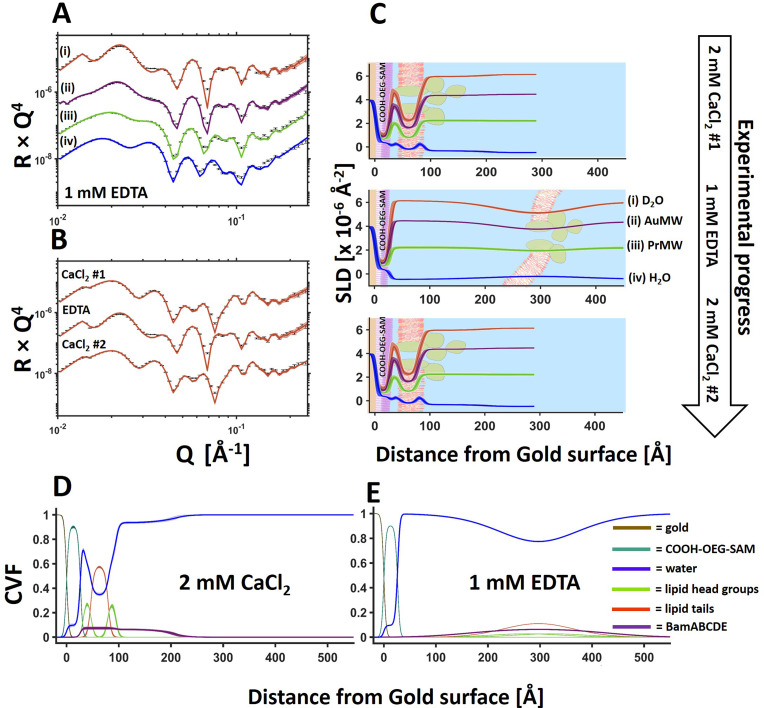
Sequestration of calcium ions by EDTA causes a reversible large-scale movement of the BamABCDE 8 : 2 (mol mol^−1^) POPC : POPS floating membranes from the COOH-OEG-SAM surface. Neutron reflectometry (error bars) and model fits (lines) are shown for a protein–lipid membrane adjacent to a COOH-OEG-SAM coated gold surface under multiple solution isotopic contrast conditions in 20 mM HEPES pH/D 7.2 1 mM EDTA (A). This sample was analysed initially in 2 mM CaCl_2_ then 1 mM EDTA and then again in 2 mM CaCl_2_. Changes to the NR data with changing solution conditions are shown for the D_2_O solution contrast in B. The changes in the distance and distribution of the membrane from the surface during this process are shown in C through the changes in the position of the membrane relative to the COOH-OEG-SAM. Changes in the position of individual components can be seen by comparing the component volume fraction plots in 2 mM CaCl_2_ (D) and 1 mM EDTA (E). The range of acceptable fits used to generate the 65% confidence intervals for the fitting parameters are shown as line width reflectivity profiles in A and B. The ambiguity in the resolved interfacial structure determined from this are shown as line widths in SLD profiles given in C and the component volume fraction profiles given in D and E.

Changing the solution salt conditions was found (like with the lipid only membranes previously^17^) to modify the floating membrane-to-surface distances. Fig. 3 shows an example of the movement of the floating membrane with respect to the COOH-OEG-SAM surface. The protein–lipid membranes were found to be in close approach to the sample surface in the presence of Ca^2+^ ions (Fig. 3D) but moved to a distance of >130 Å from the surface once the divalent cations were sequestered from the sample. At large distances from the bulk surface the fluctuational amplitude of the membrane increases,^36–38^ causing the density profile of the membrane to spread over a wide range, as can be seen in the presence of EDTA (Fig. 3C, middle and E).^17^ Reintroducing Ca^2+^ ions into the membrane bathing solution caused the bilayer to return to its previous position with no apparent change in structure (Fig. 3B and C) suggesting reversible membrane distance tuning.

Previous simulation studies suggested the close distance between the membrane and the COOH-OEG-SAM in the presence of calcium cations was due to two factors. A reduction of electrostatic repulsion between the SAM and membrane through cation binding to the anionic SAM surface and, potentially, bridging of the anionic groups present on both the carboxylate terminated SAM and the phospholipids of the membrane by the cations which accumulate in the water interlayer. Changing cations from divalent to monovalent increased repulsion leading to increases in the SAM-to-membrane distance.^17^ Altering the mono and divalent cation ratio was found to allow tuning of the SAM-to-membrane distance between the values for the membranes in the solution of either mono or divalent cations only.

**Fig. 4:**
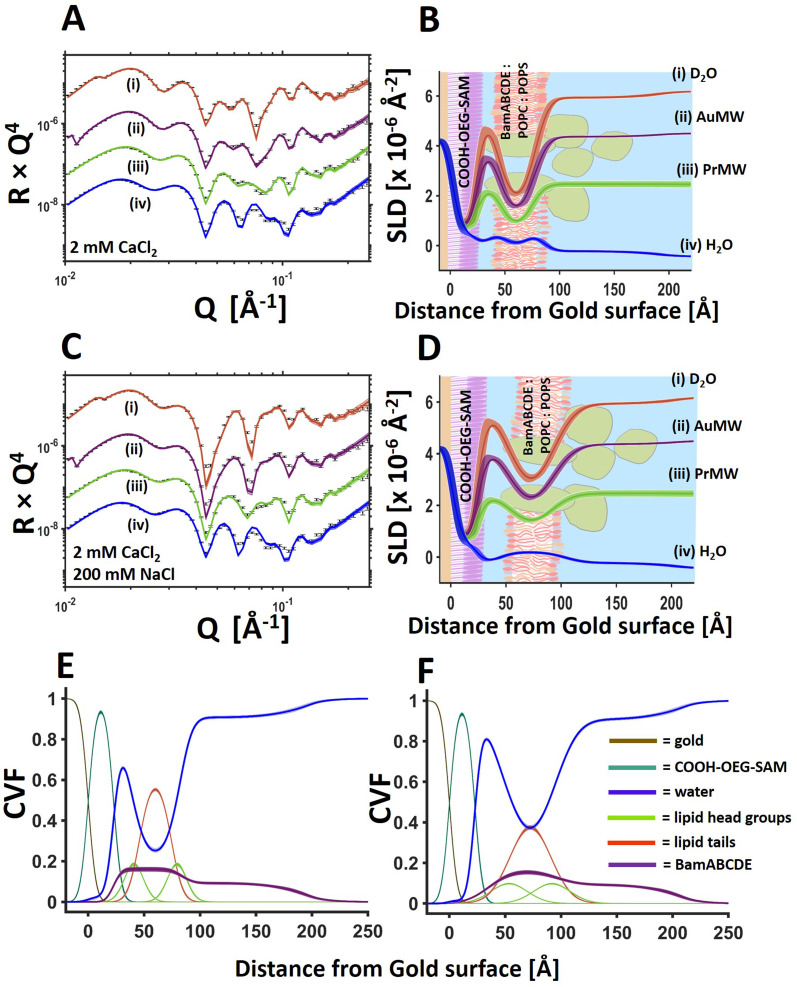
BamABCDE 8 : 2 (mol mol^−1^) POPC : POPS floating membranes change surface distance under differing solution salt conditions. NR profiles (error bars) and model data fits (lines) under multiple solution isotopic contrast conditions for a floating protein–lipid membrane sample examined sequentially in 20 mM HEPES pH/D 7.2 with 2 mM CaCl_2_ (A) and 20 mM HEPES pH/D 7.2 with 2 mM CaCl_2_ and 200 mM NaCl (C) solutions. The fit determined SLD profiles of the gold/water interfacial region are shown to the right of the relevant reflectometry profiles with a schematic of the proposed interfacial structure superimposed (B and D for 2 mM CaCl_2_ and 2 mM CaCl_2_ 200 mM NaCl respectively). The component volume fraction distributions and the changes to these upon changing solution conditions are given for 2 mM CaCl_2_ (E) and 2 mM CaCl_2_, 200 mM NaCl (F). The range of acceptable fits used to generate the 65% confidence intervals for the fitting parameters are shown as line-width reflectivity profiles in B and D. The ambiguity in the resolved interfacial structure determined from this are shown as line widths in SLD profiles given in B and D and the component volume fraction profiles given in E and F.

For the protein–lipid membranes the distance of the membrane from the bulk surface could also be fine-tuned using a combination of mono and divalent cations. Fig. 4 shows NR data from a BamABCDE POPC : POPS floating supported bilayer (the same sample as shown in Fig. 3) under solution salt conditions of 2 mM CaCl_2_ and 200 mM NaCl_._ Results revealed the SAM-to-bilayer distance increased from 11 ± 1 Å in 2 mM CaCl_2_ only to 26 ± 0.5 Å in a combination of 2 mM CaCl_2_ and 200 mM NaCl (Fig. 4 and Table 2).

**Table 2:** Floating BamABCDE POPC : POPS membrane distance from the COOH-OEG-SAM surface under differing solution electrolytic conditions

Solution	2 mM CaCl_2_	2 mM CaCl_2_	1 mM EDTA
		200 mM NaCl	
Membrane-to-SAM Distance (Å)	11 (−1, +1)	26 (−0.5, +0.5)	132 (−9, +9)
Bilayer roughness (Å)	7 (−1, +1)	16 (−1, +1)	68 (−2, +2)

**Fig. 5:**
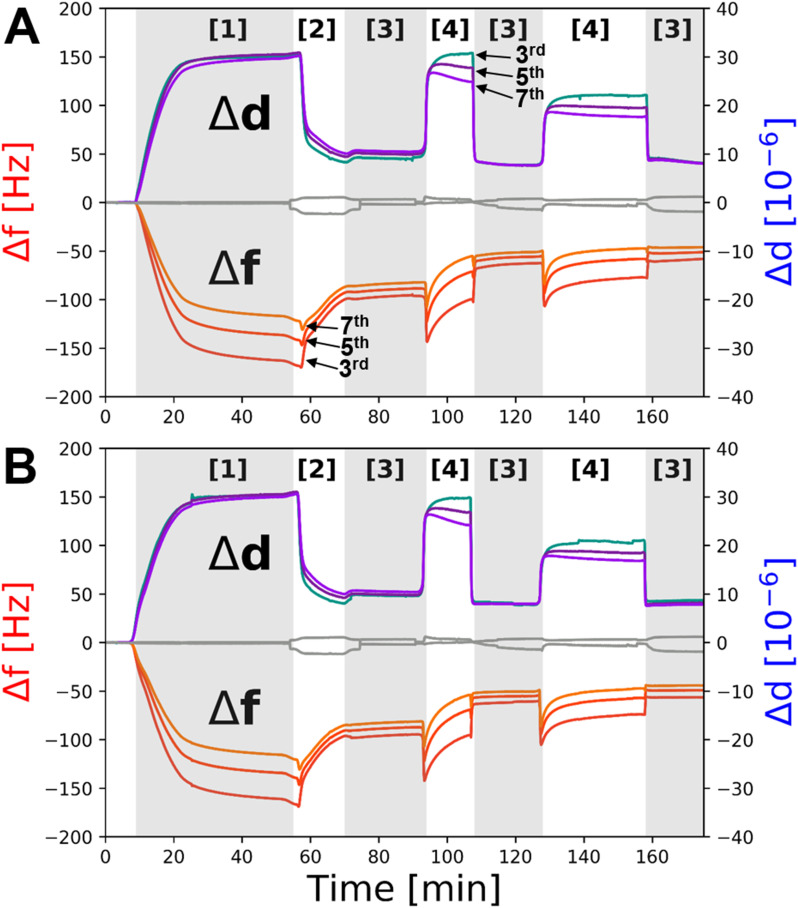
QCM-D data showing the deposition and distance tuning behaviour of BamABCDE POPC : POPS membranes adjacent to COOH-OEG-SAM coated gold surfaces. Changes in the 3^rd^, 5^th^ and 7^th^ overtones during adsorption of BamABCDE POPC : POPS vesicles onto the COOH-OEG-SAM coated gold surface [1] and their rupture by osmotic shock [2] to produce self-assembled floating supported membranes are shown for two individual sensor surfaces run in parallel (A and B). After sample fabrication the ion content in the HEPES buffer solution bathing the floating membranes was iterated between 2 mM Ca^2+^ [3] and 1 mM EDTA [4] in 20 mM Tris–HCl buffer pH 7.2 to demonstrate the reversible movement of the bilayer through changes in the mass of the water interlayer between the floating membranes and the COOH-OEG-SAM. The grey line in panels A and B is frequency and dissipation data from a COOH-OEG-SAM sensor without the presence of a protein–lipid bilayer at the SAM/water interface.

QCM-D measurements were used to assess if the deposition and distance tuning behaviour of the same Bam-lipid membrane system adjacent a COOH-OEG-SAM could be conducted on a sensor surface. The addition of BamABCDE : POPC : POPS vesicles to the QCM flow cells led to a very large decrease in resonant frequency (−170 Hz on Δ*f*_3_) and a concomitant increase in dissipation (∼30 ppm), characteristic of the deposition of a viscoelastic material onto the gold sensor surface, commonly associated with vesicle adsorption (Fig. 5 [1]). An exchange of buffer into H_2_O with 2 mM CaCl_2_, to promote osmotic shock, was performed. This resulted in an overall resonance frequency decrease to ∼70 Hz for the lipid deposition process (Fig. 5A and B [2] after first EDTA wash). This is larger than one would expect for deposition of a lipid bilayer alone (∼27 Hz on Δ*f*_3_)^29^ but is consistent with the additional mass associated with BamABCDE (∼200 kDa) and the water interlayer between the membrane and the SAM. It was interesting to note that during osmotic shock a small decrease in Δ*f* was observed before a large decrease, suggestive of initial swelling then rupturing of the SAM surface adsorbed vesicles (Fig. 5A and B [2]) which is expected for this deposition method.^39^

Next, the effect of divalent cation sequestration through the addition of EDTA was investigated. For the control, little change was observed (Fig. 5A and B, grey line). However, with BamABCDE : POPC : POPS floating membranes present on the COOH-OEG-SAM coated sensor surfaces a large increase in dissipation (∼20–30 ppm) was noted. This is highly indicative of a large change in viscoelasticity at the surface, consistent with the observed increases in amplitude of the membrane and increased spread of density noted in the NR data. The concurrent changes in Δ*f* were observed by an initial large decrease in frequency followed by a slow increase (Fig. 5A and B [4]). This was interpreted as the movement of the membrane away from the surface where it was initially still surface coupled, leading to a decrease in frequency as the mass of water between the membrane and COOH-OEG-SAM increased. However, as the membrane moved to larger distances relative to the SAM surface it partially lost its surface coupling leading to a frequency increase as the mass of membrane and the water between it and the SAM was lost. The addition of 2 mM calcium ions to the QCM-D flow cells returned the system back to a similar state prior to EDTA addition with a drop in viscoelasticity and an increase in frequency, consistent with the return of the bilayer to its previous position close to the sensor surface with a reduction in water mass between the SAM and the membrane as a result. Repeat cycling of EDTA/calcium showed reproducible behaviour with increases/decreases in Δ*f* and Δ*d* consistent with the membrane moving away from and returning close to the COOH-OEG-SAM surface respectively (Fig. 4A and B [3] to [4]). This behaviour showed the sample system was behaving in a manner comparable to that observed in the NR data. It should be noted that the Δ*f* values in 2 mM CaCl_2_ directly after membrane fabrication were lower (∼120 Hz on Δ*f*_3_) than when 2 mM CaCl_2_ was returned to the flow cells after the first EDTA rinse of the system (∼70 Hz on Δ*f*_3_) but remained consisted in the solution salt condition upon solution changes after this (see Fig. 5). This was interpreted to be due to a minor component of non-planar material present above the bilayer surface directly after vesicle rupture which was removed from the interface after the first EDTA rinse leaving only the planar protein–lipid membrane. This material was likely a small surface coverage of non-ruptured vesicles which QCM-D would be sensitive to (due to their large relative mass) but would not be easily observed in NR measurements due to their low SLD of these compared to the bulk solution (as vesicles are composed mostly of solution).

Activity testing of the floating BamABCDE : POPC : POPS membranes was undertaken to measure if the Bam complex was able to catalyse the folding of outer membrane protein T (a bacterial outer membrane porin) in the self-assembled floating supported membranes. NR results from these measurements are described in the ESI (see Fig. S1, S2† and associated text). The analysis of the NR data revealed some evidence that the Bam complex was able to catalyse Porin folding into the lipid matrix of the floating membrane. This data, though preliminary, provides support for the viability of the self-assembled floating membranes as an assay system for studies on IMPs within their native lipid matrix.

The complex IMP containing floating supported bilayers demonstrated here complements and enhances upon the capabilities of other supported lipid membranes types.^40^ The sample system has the same ease of assembly as supported lipid bilayers deposited by vesicles rupture.^41^ In addition it also offers the ability to incorporate IMPs into the planar membrane found in protein-tethered membranes^11^ as well as the biological accuracy of floating supported bilayers.^13^ This coupled with the membrane-to-surface tuneability of the sample system makes for a versatile platform for in *in vitro* studies on IMP biochemistry on a sensor surface.

## Conclusions

Here we have demonstrated that floating planar bilayers containing integral membrane proteins embedded in their native lipid environment can be fabricated onto sensor surfaces using a two-step self-assembly process. Furthermore, the distance of the resulting protein lipid complex can be tuned to the experimental requirements using millimolar changes in the solution salt conditions. This sample platform therefore represents a step forward in the accuracy and sophistication of planar *in vitro* model membrane systems allowing for biologically accurate studies of integral membrane proteins without modification to the lipid or protein components of the sample. This system presents a convenient and robust sample platform with which to undertake analytical studies on a range of integral membrane proteins in their native lipid matrix under biologically accurate sample conditions. In future this sample system could be applied to *in vitro* studies on IMP mediated biochemical processes and as a bio-sensor based assay system for the binding of drugs to target membrane proteins.

## Abbreviations

Bam: β-Barrel assembly machinery
EDTA: Ethylenediaminetetraacetic acid
IMP: Integral membrane protein
NR: Neutron reflectometry
POPC: 1-Palmitoyl-2-oleoyl-*sn-glycero*-3-phosphocholine
POPS: 1-Palmitoyl-2-oleoyl-*sn-glycero*-3-phospho-l-serine
QCM-D: Quartz crystal microbalance with dissipation
SAM: Self-assembled monolayer

## Author contributions

S.H., L.A.C. and T.K. designed research; S.H., D.H., E.C.B., H.J., R.H., T.O., P.S., T.K. and L.A.C. performed research; S.H., E.C.B. and L.A.C. analyzed data; L.A.C., S.H., E.C.B., D.H. and T.K. wrote the paper.

## Conflicts of interest

There are no conflicts to declare.

## Supplementary Material

NR-016-D3NR04622B-s001

## References

[cit1] Clifton L. A., Campbell R. A., Sebastiani F., Campos-Terán J., Gonzalez-Martinez J. F., Björklund S., Sotres J., Cárdenas M. (2020). Adv. Colloid Interface Sci..

[cit2] Whitelegge J. P. (2013). Anal. Chem..

[cit3] Uhlén M., Fagerberg L., Hallström B. M., Lindskog C., Oksvold P., Mardinoglu A., Sivertsson Å., Kampf C., Sjöstedt E., Asplund A., Olsson I., Edlund K., Lundberg E., Navani S., Szigyarto C. A.-K., Odeberg J., Djureinovic D., Takanen J. O., Hober S., Alm T., Edqvist P.-H., Berling H., Tegel H., Mulder J., Rockberg J., Nilsson P., Schwenk J. M., Hamsten M., von Feilitzen K., Forsberg M., Persson L., Johansson F., Zwahlen M., von Heijne G., Nielsen J., Pontén F. (2015). Science.

[cit4] Terstappen G. C., Reggiani A. (2001). Trends Pharmacol. Sci..

[cit5] Davey J. (2004). Expert Opin. Ther. Targets.

[cit6] Arinaminpathy Y., Khurana E., Engelman D. M., Gerstein M. B. (2009). Drug Discovery Today.

[cit7] Ratkeviciute G., Cooper B. F., Knowles T. J. (2021). Biochem. Soc. Trans..

[cit8] Kleinschmidt J. H., Popot J.-L. (2014). Arch. Biochem. Biophys..

[cit9] Lee S. C., Knowles T. J., Postis V. L. G., Jamshad M., Parslow R. A., Lin Y., Goldman A., Sridhar P., Overduin M., Muench S. P., Dafforn T. R. (2016). Nat. Protoc..

[cit10] Denisov I. G., Sligar S. G. (2016). Nat. Struct. Mol. Biol..

[cit11] Giess F., Friedrich M. G., Heberle J., Naumann R. L., Knoll W. (2004). Biophys. J..

[cit12] Hughes A. V., Holt S. A., Daulton E., Soliakov A., Charlton T. R., Steven J., Lakey J. H., Roser S. J. (2014). J. R. Soc. Interface.

[cit13] Fragneto G., Charitat T., Daillant J. (2012). Eur. Biophys. J. Biophys. Lett..

[cit14] Clifton L. A., Holt S. A., Hughes A. V., Daulton E. L., Arunmanee W., Heinrich F., Khalid S., Jefferies D., Charlton T. R., Webster J. R. P., Kinane C. J., Lakey J. H. (2015). Angew. Chem., Int. Ed..

[cit15] Charitat T., Lecuyer S., Fragneto G. (2008). Biointerphases.

[cit16] Hughes A. V., Howse J. R., Dabkowska A., Jones R. A. L., Lawrence M. J., Roser S. J. (2008). Langmuir.

[cit17] John L. H., Preston G. M., Sansom M. S. P., Clifton L. A. (2021). J. Colloid Interface Sci..

[cit18] Clifton L. A., Paracini N., Hughes A. V., Lakey J. H., Steinke N. J., Cooper J. F. K., Gavutis M., Skoda M. W. A. (2019). Langmuir.

[cit19] Kim K. H., Aulakh S., Paetzel M. (2012). Protein Sci..

[cit20] Hall S. C. L., Clifton L. A., Sridhar P., Hardy D. J., Wotherspoon P., Wright J., Whitehouse J., Gamage N., Laxton C. S., Hatton C., Hughes G. W., Jeeves M., Knowles T. J. (2021). Biophys. J..

[cit21] Roman-Hernandez G. and Bernstein H. D., in The BAM Complex, Methods in Mol Biol, 2015, vol. 1329, pp. 203–213 10.1007/978-1-4939-2871-2_16 PMC6440698 26427687

[cit22] Penfold J., Richardson R. M., Zarbakhsh A., Webster J. R. P., Bucknall D. G., Rennie A. R., Jones R. A. L., Cosgrove T., Thomas R. K., Higgins J. S., Fletcher P. D. I., Dickinson E., Roser S. J., McLure I. A., Hillman A. R., Richards R. W., Staples E. J., Burgess A. N., Simister E. A., White J. W. (1997). J. Chem. Soc., Faraday Trans..

[cit23] Webster J., Holt S., Dalgliesh R. (2006). Physica B: Condens. Matter.

[cit24] Clifton L. A., Ciesielski F., Skoda M. W. A., Paracini N., Holt S. A., Lakey J. H. (2016). Langmuir.

[cit25] Hughes A. V. , RasCAL, Version 1, 2019

[cit26] Born M. and Wolf E., Principles of Optics, Cambridge University Press, 1999

[cit27] Haario H., Laine M., Mira A., Saksman E. (2006). Stat. Comput..

[cit28] Sivia D. S. and Skilling J., Data analysis: a Bayesian tutorial, Oxford University Press, Oxford, UK, 2nd edn, 2006

[cit29] Nielsen S. B. and Otzen D. E., in Lipid-protein interactions. Methods in Molecular Biology, ed. J. H. Kleinschmidt, 2nd edn, 2019, pp. 31–52 10.1007/978-1-4939-9512-7_2 31218612

[cit30] Keller C. A., Glasmästar K., Zhdanov V. P., Kasemo B. (2000). Phys. Rev. Lett..

[cit31] Easley A. D., Ma T., Eneh C. I., Yun J., Thakur R. M., Lutkenhaus J. L. (2022). J. Polym. Sci..

[cit32] Clifton L. A., Paracini N., Hughes A. V., Lakey J. H., Steinke N.-J., Cooper J. F. K., Gavutis M., Skoda M. W. A. (2019). Langmuir.

[cit33] Noinaj N., Kuszak A. J., Gumbart J. C., Lukacik P., Chang H., Easley N. C., Lithgow T., Buchanan S. K. (2013). Nature.

[cit34] Cooper B. F., Ratkevičiūtė G., Clifton L. A., Johnston H., Holyfield R., Hardy D. J., Caulton S. G., Chatterton W., Sridhar P., Wotherspoon P., Hughes G. W., Hall S. C., Lovering A. L., Knowles T. J. (2024). EMBO Rep..

[cit35] Han L., Zheng J., Wang Y., Yang X., Liu Y., Sun C., Cao B., Zhou H., Ni D., Lou J., Zhao Y., Huang Y. (2016). Nat. Struct. Mol. Biol..

[cit36] Michalak D. J., Lösche M., Hoogerheide D. P. (2021). Langmuir.

[cit37] Mecke K. R., Charitat T., Graner F. (2003). Langmuir.

[cit38] Daillant J., Bellet-Amalric E., Braslau A., Charitat T., Fragneto G., Graner F., Mora S., Rieutord F., Stidder B. (2005). Proc. Natl. Acad. Sci. U. S. A..

[cit39] Jackman J. A., Choi J.-H., Zhdanov V. P., Cho N.-J. (2013). Langmuir.

[cit40] Sackmann E. (1996). Science.

[cit41] Hardy G. J., Nayak R., Zauscher S. (2013). Curr. Opin. Colloid Interface Sci..

