# Supplementary material for: Distance tuneable integral membrane protein containing floating bilayers via in situ directed self-assembly
Source: Nanoscale. 2024 Jun 24;16(28):13503–15. doi: 10.1039/d3nr04622b (PMC11256219; doi:10.1039/d3nr04622b)
Supplement: NR-016-D3NR04622B-s001 [file NR-016-D3NR04622B-s001.pdf]

Electronic Supplementary Material (ESI) for Nanoscale.
This journal is © The Royal Society of Chemistry 2024

Supporting Information

Distance Tuneable Integral Membrane Protein Containing Floating Bilayers via In Situ Directed Self-Assembly

Stephen C. L. Hall[1], David Hardy[2], Éilís C. Bragginton[3], Hannah Johnston[2], Tudor. Onose[2], Rachel Holyfield[2], Pooja Sridhar[2], Timothy Knowles[2] and Luke A. Clifton[1]*

1. ISIS Pulsed Neutron and Muon Source, Science and Technology Facilities Council, Rutherford Appleton Laboratory, Harwell Science and Innovation Campus, Didcot, Oxfordshire, OX11 OQX, UK.

2. School of Biosciences, University of Birmingham, Birmingham, B15 2TT, U.K.

3. Electron Bio-Imaging Centre (eBIC), Diamond Light Source Ltd, Diamond House, Harwell Science and Innovation Campus, OX11 0DE, UK.

*Corresponding Author - Email: luke.clifton@stfc.ac.uk

Activity Testing the BamABCDE : POPC : POPS Floating Supported Bilayers: To test if the Barrel Assembly Machinery was functional as a site for the folding of β-barrel proteins in the self-assembled floating supported membranes neutron reflectometry was employed to examine the change in structure across the bilayer before and after protein folding. A self-assembled BamABCDE : POPC : POPS bilayer was deposited adjacent a COOH-OEG-SAM coated gold surface. Once the formation of the membrane was verified the chaperone protein SurA and unfolded (u-)Outer membrane protein T (OmpF), an unfolded beta barrel porin protein, were added into the buffer solution in the solid liquid flow cell containing the floating protein-lipid bilayer. Figure S1 shows the neutron reflectometry profiles, model data fits and resulting scattering length density profiles obtained prior to and at the equilibrium interaction of SurA:OmpT with the BamABCDE: POPC : POPS bilayer.

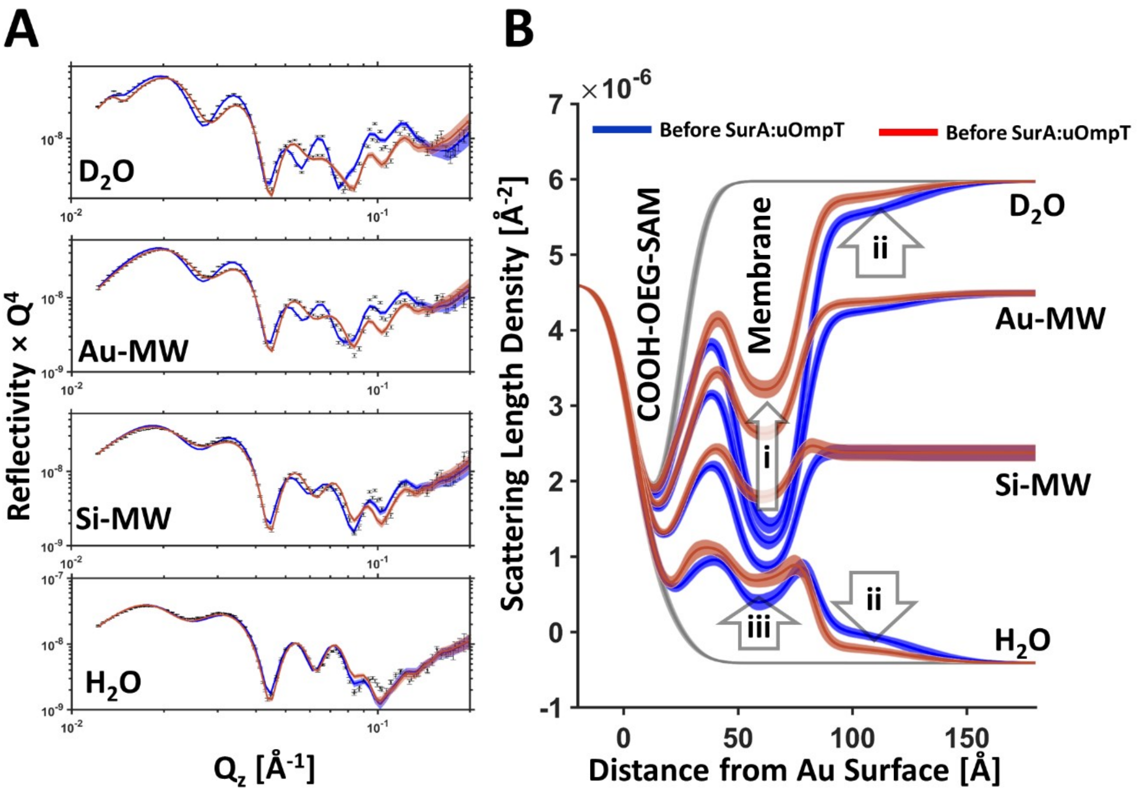

Figure S1. Neutron reflectometry profiles (error bars) and model data fits (lines) A and the scattering length density profiles these fits represent from a BamABCDE: POPC : POPS self-assembled floating bilayer adjacent a COOH-OEG-SAM coated gold surface before (blue) and after (red) the interaction of SurA:uOmpT with the floating bilayer. The range of acceptable fits used to generate the 65% confidence intervals for the fitting parameters are shown as a line width in A and the ambiguity in the resolved interfacial structure determined from this are shown as line widths in B.

Analysis of the experimental reflectometry profiles suggested two processes occurred during the interaction of SurA:uOmpT with the floating bilayer. Firstly, a decrease in the volume fraction of phospholipid and BamABCDE in the floating bilayer. The loss of phospholipids in the bilayer is emphasized in the comparative SLD plots in Fig S1 B, by arrow i, which highlights the increase in the difference in SLD of the lipid tails region of the bilayer under differing solution contrast conditions. A decrease in the BamABCDE content of the bilayer is suggested by a reduction in the coverage of the membrane surface region of the protein, highlighted in figure S1 by arrow ii. Conversely the amount of protein within the membrane was found to increase, which was noted from the increase in lipid tails SLD in all solution isotopic contrasts, highlighted in figure S1 by arrow iii.

Porin proteins are predominantly located within lipid bilayers[1] whereas the Bam complex is composed of both protein regions within the bilayer (predominantly BamA[2]) and region on the membrane surface (BamBCDE[3]). The loss of protein on the membrane surface and the loss of phospholipid during the inter action of SurA:uOmpT and the increase in protein within the bilayer suggests that as SurA delivers uOmpT to the Bam complex which is then folded into the BamABCDE : POPC : POPS floating supported bilayer. The membrane is disrupted due to the addition of material to a planar bilayer which already covers the vast majority of the sample surface. This disruption would likely be in the form of “blebbing” followed by dissociation of part of the membrane due to the presence of more material within the membrane than the total amount needed to cover the planar surface. This results in defects across the planar surface which result in an increase in solution across the membrane region in the z-direction, and a loss of BamABCDE and lipid from this. The increase in protein within the floating bilayer is due to the inclusion of folded OmpT within the floating bilayer (which is also likely add to the solution content due to its pore).

Based on this assessment of the experimental data it could be suggested that BamABCDE is active in the self-assembled floating supported membranes as a porin folding complex. Another interpretation of this data is that SurA:uOmpT acts as a membrane disrupting agent, however, this would not explain why the membrane is in the same confirmation post interaction or why an increase in protein within the bilayer region of the floating protein-lipid bilayer is observed.

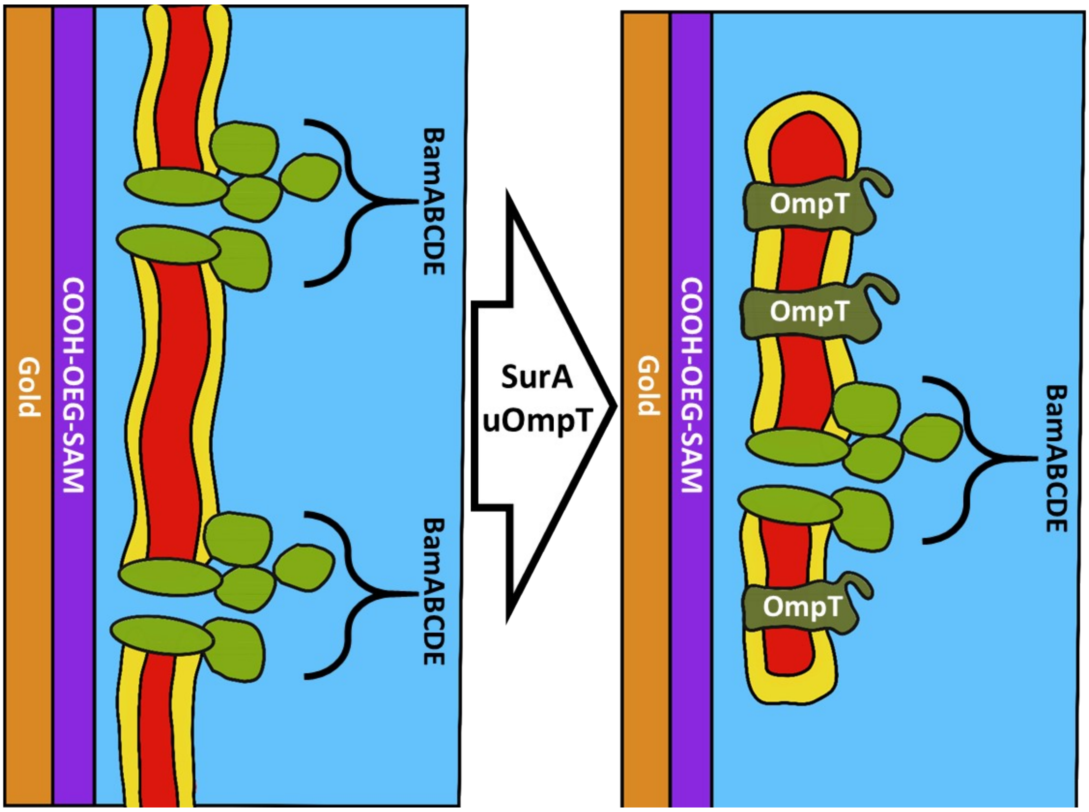

Figure S2. A schematic representation describing an interpretation of the changes in the interfacial structure of BamABCDE:POPC:POPS floating bilayers as a result of the interaction of SurA:uOmpT with the membrane.

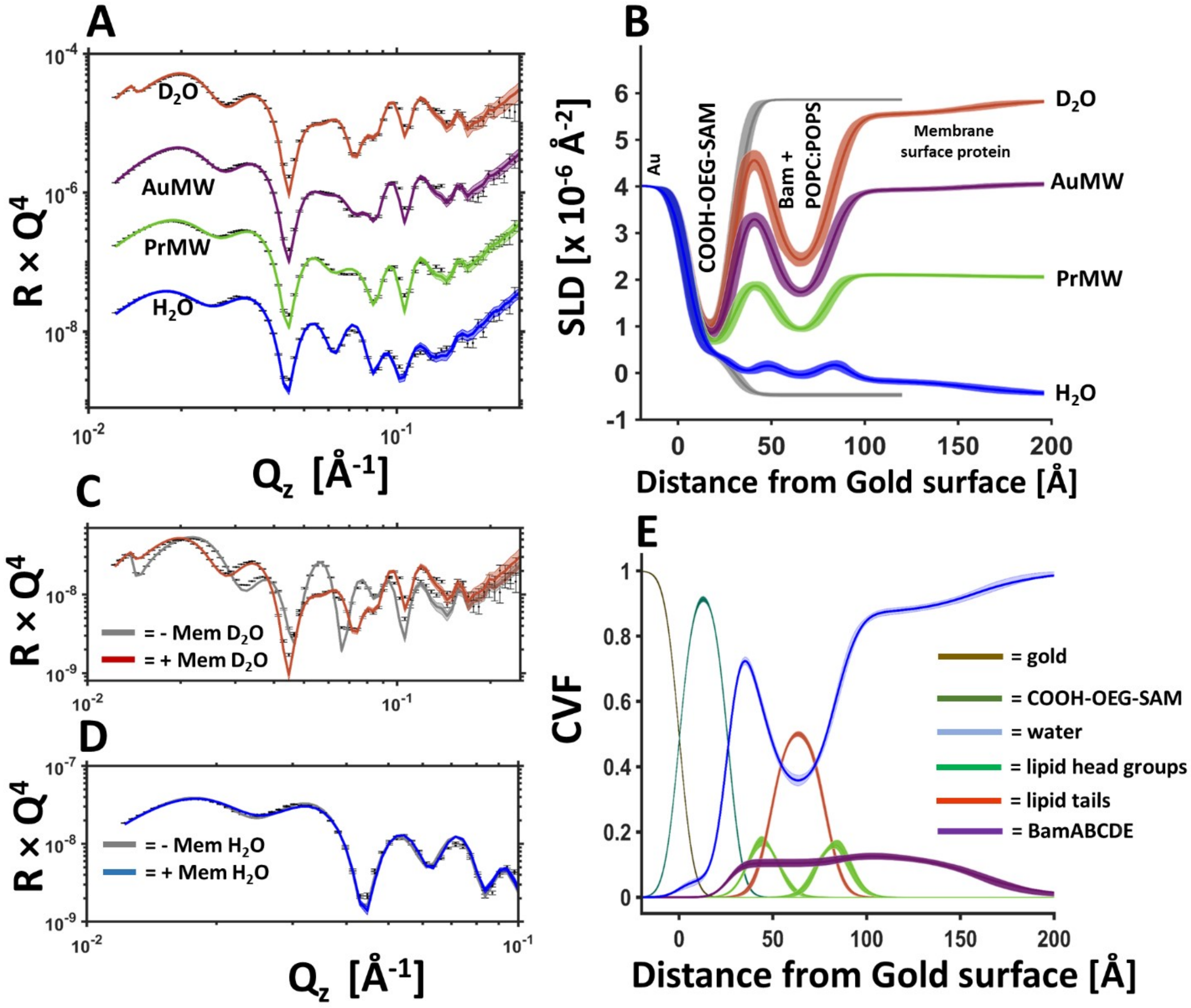

Figure S3. Repeat data. BamABCDE in a floating 8:2 (mol/mol) POPC:POPS Bilayer adjacent to a COOH-OEG-SAM at the gold/water interface. Neutron reflectometry (NR) profiles (error bars) and model data fits (lines) under multiple solution isotopic contrast conditions for the protein-lipid membrane containing sample (A) and the scattering length density profiles of the gold/water interfacial region are shown (B). A comparison of the NR profiles before and after the deposition of the floating membrane in D_2O (C) and H_2O (D) are shown to highlight the modulation of the NR profile by the membrane. Finally, the component volume fraction profile of the gold water interfacial region determined by NR data analysis is given, showing the relative distribution of the SAM, water, lipid and protein components (E). The range of acceptable fits used to generate the 65% confidence intervals for the fitting parameters are shown as a line width in A, C and D and the ambiguity in the resolved interfacial structure determined from this are shown as line widths in B and E.

Table S1, resolved structural parameters from the self-assembled floating 8:2 POPC : POPS bilayer with embedded BamABCDE.

Layer	Thickness	Composition	Roughness
**COOH-OEG-SAM**	26.0 (-0.3, +0.4) Å	95 (-1, +1) % SAM<br>5 (-1, +1) % Solution	6.2 (-0.2, +0.2) Å
**Solution Interlayer**	13.9 (-1.1, +1.2) Å	100% Solution	
**Inner Head groups**	7.3 (+ 1.0, -1.0) Å	11 (-1, +1,) % Protein<br>37 (-3, +4) % Lipid<br>52 (-4, +3) % Solution	8.0 (+1.0, -1.0) Å
**Tails**	28.0 (-0.7, +0.7) Å	11 (-1, +1,) % Protein<br>56 (-3, +3) % Lipid<br>34 (-3, +2) % Solution	
**Outer Head groups**	7.3 (+ 0.7, -0.7) Å	11 (-1, +1,) % Protein<br>37 (-3, +4) % Lipid<br>52 (-4, +3) % Solution	
**Peripheral Protein**	74.2 (-5.9, +6.0) Å	13 (-1, +1) % Protein<br>87 (-1, +1) % Solution	24.7 (-4.0, +3.4) Å

Table S2, A comparison of the resolved structural parameters for the membrane from two independent depositions of self-assembled floating 8:2 POPC : POPS bilayer with embedded BamABCDE both in 20mM HEPES pH/D 7.2 100 mM NaCl 2mM $CaCl_2$.

	Sample 1 (Fig 1 Main Article)		Sample 2 (Fig S3, supporting information)	
**Layer**	**Thickness**	**Composition**	**Thickness**	**Composition**
**Inner Head groups**	8.0 (+ 0.6, -0.6)	24 (-1, +1,) % Protein<br>42 (-3, +4) % Lipid<br>34 (-4, +3) % Solution	7.3 (+ 1.0, -1.0) Å	11 (-1, +1,) % Protein<br>37 (-3, +4) % Lipid<br>52 (-4, +3) % Solution
**Tails**	30.0 (-0.5, +0.5) Å	24 (-1, +1,) % Protein<br>61 (-2, +2) % Lipid<br>15 (-2, +2) % Solution	28.0 (-0.7, +0.7) Å	11 (-1, +1,) % Protein<br>56 (-3, +3) % Lipid<br>34 (-3, +2) % Solution
**Outer Head groups**	8.0 (+ 0.6, -0.6) Å	24 (-1, +1,) % Protein<br>42 (-3, +4) % Lipid<br>34 (-4, +3) % Solution	7.3 (+ 0.7, -0.7) Å	11 (-1, +1,) % Protein<br>37 (-3, +4) % Lipid<br>52 (-4, +3) % Solution
**Peripheral Protein**	50.5 (-3.7, +3.5) Å	13 (-1, +1) % Protein<br>87 (-1, +1) % Solution	74.2 (-5.9, +6.0) Å	13 (-1, +1) % Protein<br>87 (-1, +1) % Solution

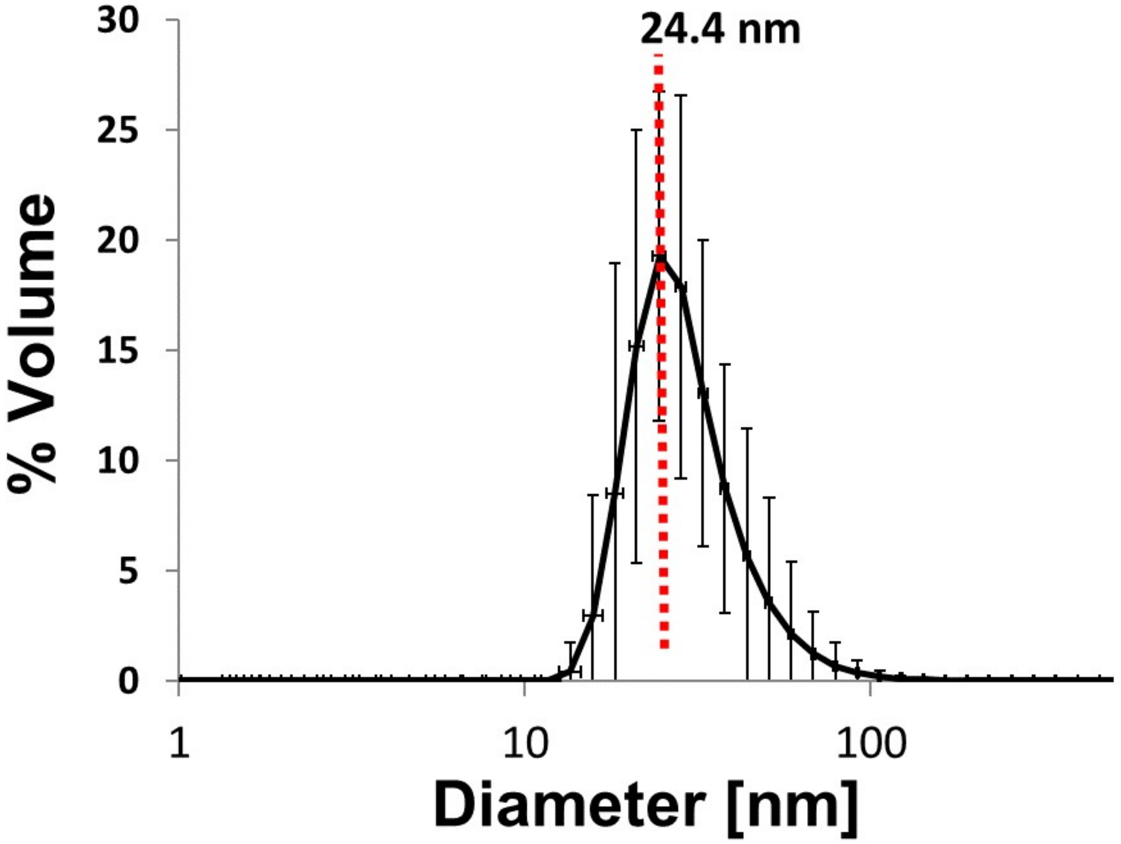

Figure S4, Dynamic light scattering data shown as particle diameter *vs.* percentage volume for the BamABCDE : 8:2 (mol/mol) POPC : POPS vesicles used to produce the self-assembled floating supported membranes.

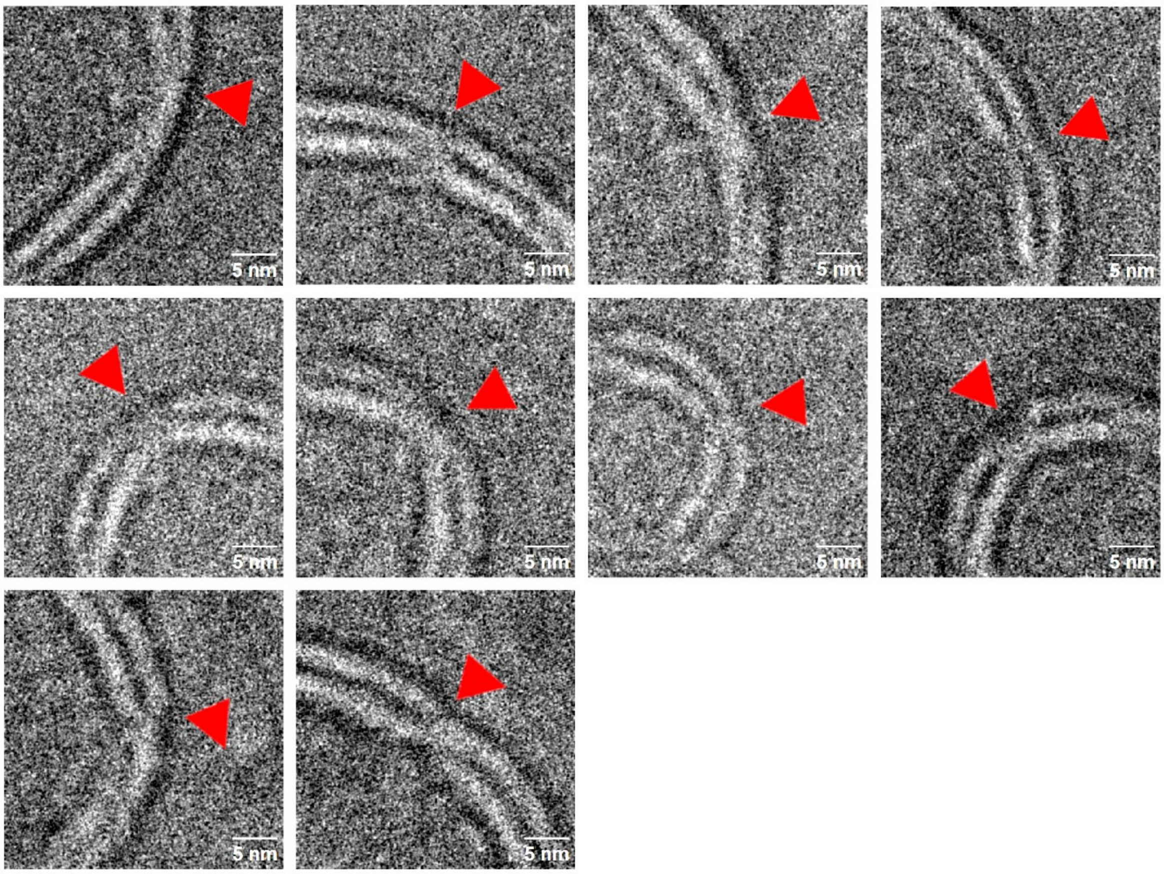

Figure S5: Examples of observed Bam complexes within proteoliposomes. In an attempt to identify the orientation of the Bam complex within proteoliposome with cryo-EM, we looked for changes in pixel intensity either side of the bilayer. In the majority of cases, the orientation was not clear. From 226 micrographs, we manually inspected and identified the presence of the Bam complex on 232 occasions of which 11 particles were observed to have differential pixel intensity on one side of the proteoliposome (4.7 %).

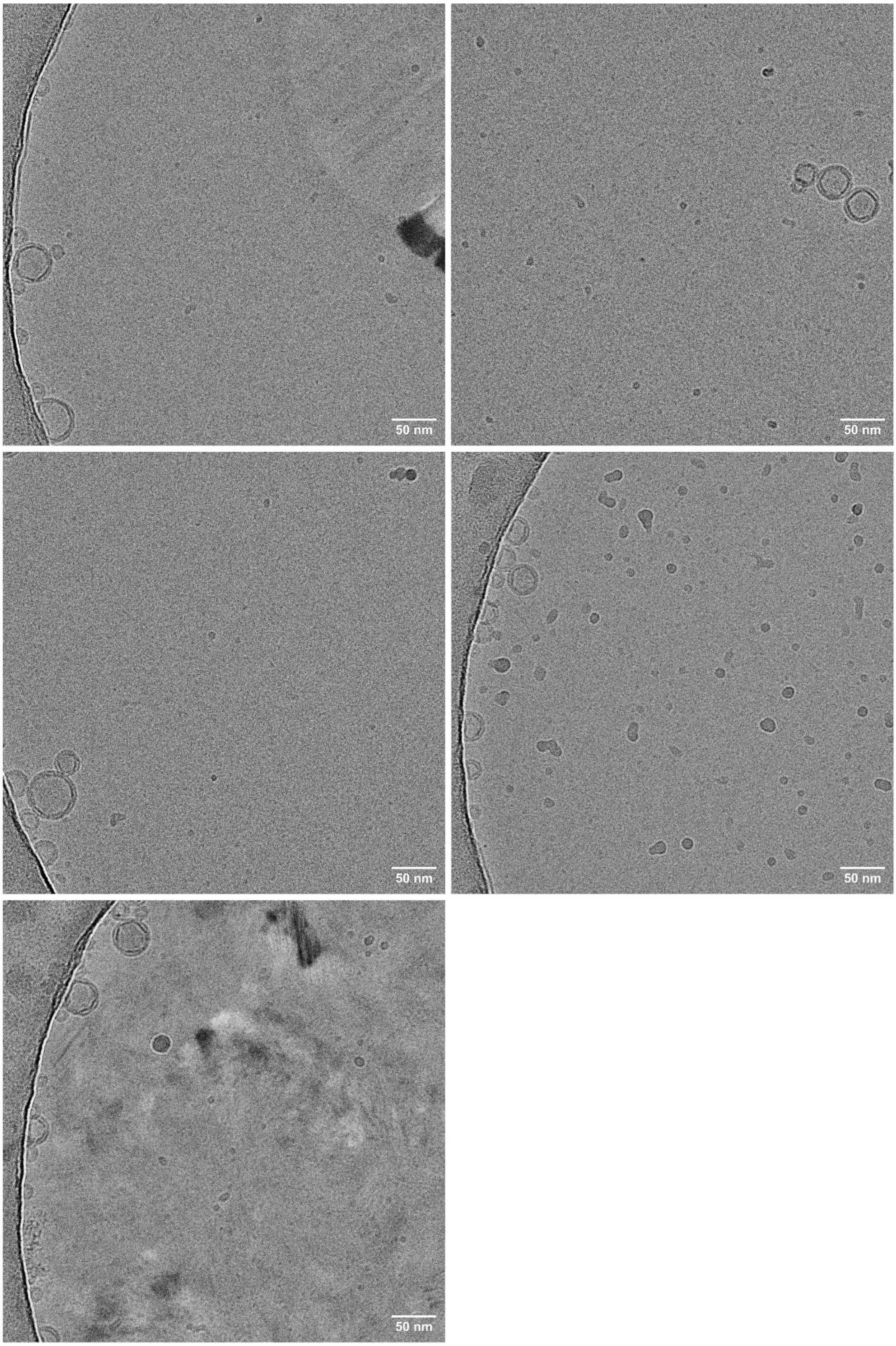

Figure S6: Uncropped micrographs of vesicles featured in Figure 2.

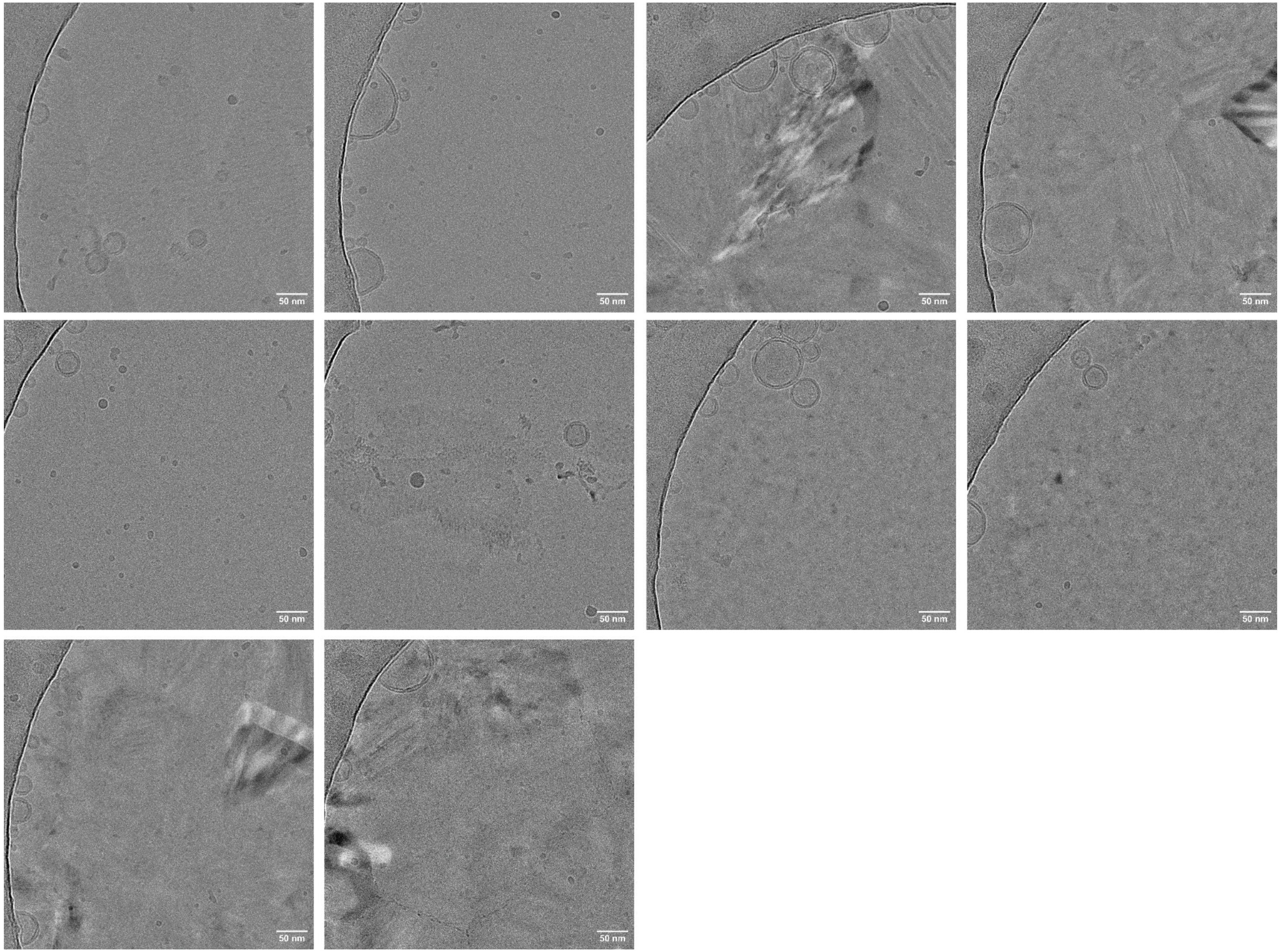

Figure S7: Uncropped micrographs of vesicles featured in figure S5.

References

1 S. Galdiero, A. Falanga, M. Cantisani, R. Tarallo, M. Elena Della Pepa, V. D'Oriano and M. Galdiero, *Curr. Protein Pept. Sci.*, 2012, **13**, 843–854.

2 J. Bakelar, S. K. Buchanan and N. Noinaj, *Science (80-.).*, 2016, **351**, 180–186.

3 T. J. Knowles, A. Scott-Tucker, M. Overduin and I. R. Henderson, *Nat. Rev. Microbiol.*, 2009, **7**, 206–214.
